# Thrombocytopenia in Critically Ill Children: A Review for Practicing Clinicians

**DOI:** 10.3390/children12010083

**Published:** 2025-01-12

**Authors:** Balagangadhar R. Totapally, Abhinav Totapally, Paul A. Martinez

**Affiliations:** 1 Division of Critical Care Medicine, Nicklaus Children’s Hospital, 3100 SW 62nd Avenue, Miami, FL 33155, USA; abhinav.totapally@nicklaushealth.org (A.T.); paul.martinez@nicklaushealth.org (P.A.M.); 2 Herbert Wertheim College of Medicine, Florida International University, Miami, FL 33199, USA

**Keywords:** thrombocytopenia, sepsis, DIC

## Abstract

Thrombocytopenia frequently occurs in patients before, during, and after admission to Pediatric Intensive Care Units (PICUs). In critically ill children, it is often due to multifactorial causes and can be a sign of significant organ dysfunction. This review summarizes the potential causes/mechanisms of thrombocytopenia in acutely ill children, their identification, and treatments, with special attention paid to septic patients. The mechanisms of thrombocytopenia include decreased production and sequestration, but the most common reason is increased destruction or consumption. This review specifically reviews and compares the presentation, pathogenesis, and treatment of disseminated intravascular coagulation (DIC) and the thrombotic microangiopathic spectrum (TMA), including thrombocytopenia-associated multiorgan failure (TAMOF), hemolytic uremic syndrome, and other diagnoses. The other etiologies discussed include HLH/MAS, immune thrombocytopenia, and dilutional thrombocytopenia. Finally, this review analyzes platelet transfusions, the various thresholds, and complications.

## 1. Mechanisms of Thrombocytopenia

Thrombocytopenia is defined as a platelet count < 150 × 10^9^/L. It is graded as mild with a platelet count of 100 to <150 × 10^9^/L, moderate with 50 to <100 × 10^9^/L, and severe with <50 × 10^9^/L [1]. The frequency of thrombocytopenia in patients admitted to the intensive care units (ICU) varies based on age, case mix, and the definition of thrombocytopenia (less than 50 × 10^9^/L to less than 150 × 10^9^/L) [1,2,3]. Thrombocytopenia is relatively common in critically ill children and is frequently present in those with sepsis. Thrombocytopenia in critically ill patients is associated with increased complications and mortality rates [4]. Although low platelets are independently associated with disturbed host response in sepsis, it is unclear whether thrombocytopenia is a marker of the disease process or is involved causally in worse outcomes [5]. In children with sepsis, thrombocytopenia can be attributed to multiple overlapping mechanisms and causes. Sepsis may occur in patients who have pre-existing thrombocytopenia or conditions that lead to thrombocytopenia. In addition, thrombocytopenia can occur in critically ill patients unrelated to the process of sepsis. The specific treatments of patients with thrombocytopenia may differ based on the underlying mechanisms of thrombocytopenia. This review summarizes the potential causes and mechanisms of thrombocytopenia in acutely ill children, their identification, and treatments.

**Figure 1:**
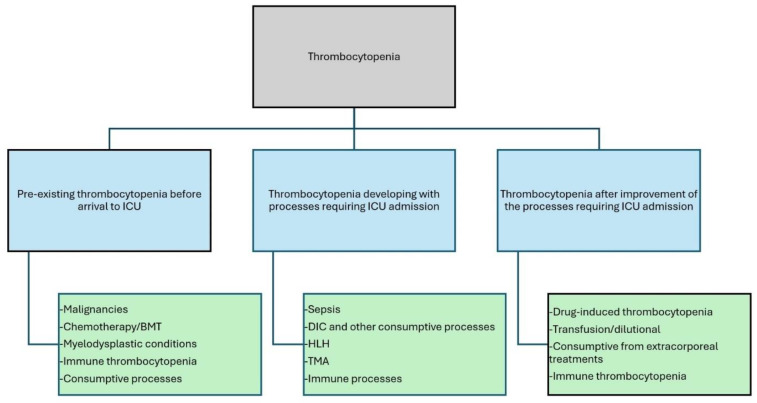
Mechanisms of thrombocytopenia in relationship to the onset of thrombocytopenia in critically ill children. BMT = bone marrow transplantation; HLH = hemophagocytic lymphohistiocytosis; TMA = thrombotic microangiopathy.

Thrombocytopenia in patients with sepsis or critical illness is multifactorial. The mechanisms of thrombocytopenia include decreased production, sequestration, increased consumption, or increased destruction. Thrombocytopenia is more commonly caused by inflammation from sepsis or sepsis-like conditions (burns, pancreatitis, or multiple trauma). However, it can also occur from pre-existing bone-marrow-suppressing conditions, such as malignancies, cancer chemotherapy, transplantation, or from medications used in the ICU, or other miscellaneous causes. Multiple mechanisms may exist in a given patient (Figure 1).

### 1.1. Spurious Thrombocytopenia, or Pseudothrombocytopenia

The first step in diagnosing thrombocytopenia is ensuring that the laboratory results reflect the actual platelet counts. Spurious thrombocytopenia, known as pseudothrombocytopenia, occurs when platelets clump together in blood collection tubes. This clumping is typically caused by ethylenediaminetetraacetic acid (EDTA)-dependent antibodies or an insufficient amount of anticoagulant [1,6]. This is most commonly observed in patients with sepsis, autoimmune disease, malignancies, or liver disease [7]. Measuring platelets using autoanalyzers will report low counts in such situations. If pseudothrombocytopenia is suspected, visual inspection of the blood smear for platelet clumps and manual platelet counts will help to differentiate.

### 1.2. Decreased Production

Decreased platelet production can occur in patients with pre-existing bone marrow suppression (malignancies, chemotherapy, and other medications). Bone marrow suppression, including thrombocytopenia, can occur with certain viral infections [8,9]. Decreased production is not the primary mechanism in most patients with sepsis without oncological premorbid conditions. Although inflammatory cytokines may depress erythropoiesis, they stimulate thrombopoiesis [10]. Hence, we observe thrombocytosis in many patients with infections as a marker of inflammation.

### 1.3. Sequestration

Sequestration of platelets in the portal circulation and spleen is an uncommon mechanism of thrombocytopenia that can occur in patients with portal hypertension and sickle cell disease [11]. Platelet sequestration may increase platelet resistance following platelet transfusion [12]. In hypersplenism, there is increased destruction of platelets from an enlarged, congested spleen, typically observed with portal hypertension [13,14].

### 1.4. Destruction, Consumption, or Both

The most common mechanisms for thrombocytopenia in critically ill patients are destruction or consumption. While both terms are often used interchangeably, “consumption” of platelets refers to being actively used up during blood clotting or in response to injury. In contrast, “destruction” of platelets implies a more targeted breakdown of platelets, often by the immune system, where antibodies attack and destroy them, decreasing platelet count in the blood. Immune or non-immune mechanisms can cause platelet destruction or consumption. Several intermediary processes are vital in platelet activation, destruction, or consumption in various disease states. Multiple simultaneous or overlapping mechanisms may be involved in the development of thrombocytopenia in any patient, especially with sepsis. Various intermediary processes involved in platelet destruction or consumption are listed below.

Thrombin: Thrombin is generated as a physiological mechanism after any tissue injury and inflammation. Excessive and uncontrolled thrombin generation after trauma or surgery or with infection can lead to the consumption of platelets along with other coagulation factors, resulting in disseminated intravascular coagulation (DIC).

Antibodies: Immune-mediated thrombocytopenia is a less common but important mechanism of low platelets in patients in the ICU. Several medications (ranitidine, beta-lactams, vancomycin, and linezolid, etc.) administered to critically ill patients can lead to immune-mediated thrombocytopenia. Autoantibodies (immune thrombocytopenia (ITP)) and alloantibodies (post-transfusions) are other less common mechanisms of immune-mediated thrombocytopenia. Heparin-induced thrombocytopenia (HIT) can occur in patients exposed to heparin due to the development of antibodies against the heparin–PF4 complex.

Hemophagocytosis: Uncontrolled cytokine production and unregulated macrophage activation can lead to hemophagocytic lymphohistiocytosis or macrophage activation syndrome (HLH/MAS) with cytopenias associated with infections, malignancies, or autoimmune processes [15]. HLH/MAS is an increasingly recognized mechanism for thrombocytopenia in critically ill patients.

Histones: Release of extracellular histones as a part of neutrophil extracellular traps (NETs) leads to platelet aggregation and thrombocytopenia. Critical illness and sepsis are associated with increased plasma histone levels and low platelets [16,17].

A Disintegrin and Metalloproteinase with Thrombospondin Type 1 Motif, Member 13 (ADAMTS13) Depletion: In thrombotic thrombocytopenia (TTP), ADAMTS13 activity is <10%, which leads to decreased clearance of Von Willebrand factor (VWF) macro-aggregates with resultant platelet aggregation and thrombocytopenia. Sepsis is also associated with low ADAMTS13 activity, although not as low as in TTP (≥10%). Plasma exchange has been used to manage thrombocytopenia associated with low ADAMTS13 in septic patients.

Complement Activation: The classical complement pathway may be activated by immune complexes, and the alternate pathway may be activated by the bacterial cell wall, leading to the formation of the terminal membrane attack complex (MAC). The MAC is a potent platelet activator and contributes to platelet destruction. Atypical and secondary thrombotic microangiopathy (aTMA and sTMA) may lead to thrombocytopenia in critically ill patients with sepsis and other TMA-predisposing conditions. Anti-complement medications may be used in their management if complement activation is the mechanism for TMA.

## 2. Thrombocytopenia Patterns

The trajectory of thrombocytopenia in critically ill patients after their admission to an ICU may provide some etiologic and prognostic information. Zarychanski et al. described the platelet trajectories and their clinical patterns [4] as follows:

1. Admitted with low platelets, remaining low during ICU stay: Thrombocytopenia may be due to a cause independent of the acute illness, such as bone marrow failure, chemotherapy, hypersplenism, ITP, etc.
2. Platelets fall immediately after admission and recover quickly: This pattern is typically observed after major surgery, cardiopulmonary bypass, and massive transfusion.
3. Platelets fall within a few days after admission and recover with improvement in critical illness: This is a pattern observed with septic patients and other major inflammatory conditions such as burns and pancreatitis. Typically, platelets fall to a nadir within two days and stay low for six days in patients with sepsis.
4. Platelets fall in a few days after admission and remain low even with the patient recovering from critical illness: Consider immune-related thrombocytopenia, like HIT, drug-induced thrombocytopenia, and transfusion-related thrombocytopenia.
5. Platelets fall within a few days and remain low with multiple organ failure: This pattern is observed in the sickest patients and is associated with a high mortality rate. Multiple phenotypes with hyperinflammation, such as Thrombocytopenia-Associated Multiple Organ Failure (TAMOF), DIC, HLH/MAS, and TMA, can be observed in patients with sepsis and thrombocytopenia.

## 3. Clinical Conditions with Thrombocytopenia

### 3.1. Disseminated Intravascular Coagulation (DIC)

**Figure 2:**
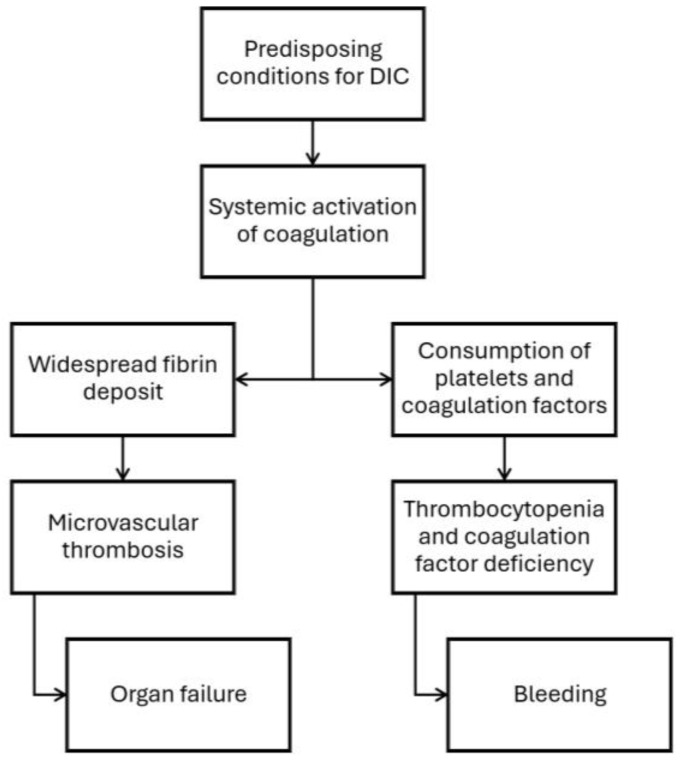
Mechanisms of organ failure and bleeding in patients with disseminated intravascular coagulation.

The immune and coagulation systems are activated in response to sepsis, severe trauma, burns, pancreatitis, and other severe systemic inflammatory conditions [5]. Inflammatory changes often lead to endothelial injury, triggering the release of tissue factor (TF), a potent coagulation cascade initiator [16]. DIC is the most common mechanism attributed to thrombocytopenia in patients with sepsis [18]. In sepsis, activation of the coagulation system is a continuum, and the extreme end is DIC. Even in the absence of overt DIC, the markers of coagulation activation are frequently present in patients with sepsis and low platelets. The milder form of activated coagulation in patients with sepsis is called sepsis-induced coagulopathy (SIC) [19,20]. SIC is characterized by systemic inflammation and early coagulation activation [17]. Tissue-factor-initiated coagulation cascade activation leads to thrombin generation, widespread coagulation, impaired anticoagulation mechanisms, and microvascular thrombosis, leading to organ ischemia and failure [17]. Consumption of platelets and coagulation factors may lead to bleeding at various sites (Figure 2) [21].

DIC diagnosis should occur with clinical conditions predisposed to DIC, along with laboratory findings. DIC is a dynamic process, and serial laboratory test measurements may be helpful [21]. No one laboratory test is specific for diagnosing DIC. Several scoring systems have been used to make diagnoses [18]. The International Society of Thrombosis and Haemostasis (ISTH) developed a scoring system that is widely used and helpful in children [22,23]. The ISTH score detects overt DIC and is not sufficiently sensitive to detect mild coagulation abnormalities associated with SIC. A scoring system to detect SIC for early detection of coagulation abnormalities has been reported [19]. Other well-accepted scoring systems for DIC diagnosis include the Japanese Ministry of Health and Welfare (JMHW) criteria [24], the Japanese Association of Acute Medicine (JAAM) criteria [25], and the Japanese Society on Thrombosis and Hemostasis (JSTH) criteria for infection-associated DIC [26].

Although not specific, thrombocytopenia is the most sensitive assay for diagnosing DIC [21]. The abnormal laboratory tests in DIC, in order of their frequency, are thrombocytopenia, elevated fibrin degradation products (FDPs), prolonged prothrombin time (PT), prolonged activated partial thromboplastin time (aPTT), and decreased fibrinogen [21]. FDPs and D-dimers (DD) may be increased in other conditions without DIC. Prolonged PT and aPTT are due to the consumption of coagulation factors in DIC, but they may be pronged due to liver dysfunction, vitamin K deficiency, or dilutional coagulation factor deficiency in massive transfusion. Nearly half the patients with DIC will have normal or even shorter PT and aPTT. Hence, the absence of prolonged PT and aPTT does not rule out DIC. Fibrinogen, an acute-phase protein, may not be reduced until DIC is severe, and it is typically not decreased with SIC. The fibrinogen levels are normal in the early stages, and mild forms of DIC and serial measurements may be useful. Fragmented red blood cells are observed with <10% of RBCs, and many fragmented RBCs indicate a microangiopathic process. In addition, the antithrombin III and protein C levels may be decreased. Thromboelastography (TEG) or rotational thromboelastometry (ROTEM) may be useful as a screening tool in DIC and SIC [27]. In DIC, maximum clot firmness (MCF) is reduced from either low platelets or low levels of fibrinogen. However, it is unknown if this is sensitive to detect early changes in coagulation during the DIC process [28]. In an observational study in adults, the lysis index with ROTEM was able to discriminate between patients with severe sepsis and postoperative patients [29]

Activation of the fibrinolytic pathway is common with DIC. However, DIC can be classified as hyperfibrinolytic or hypofibrinolytic. In hyperfibrinolytic DIC, the de novo fibrinolytic mechanisms are activated, and decreased fibrinogen is common and associated with bleeding complications. This type of DIC is mostly observed in patients with multi-trauma. In these patients, factor replacement with FFP, cryoprecipitate, platelet, and antifibrinolytic agents such as tranexamic acid may be useful [30]. In sepsis, the DIC is hypofibrinolytic, and thrombosis predominates due to the inhibition of plasminogen activator inhibitor-1 (PAI-1) with inflammatory cytokines [31]. The laboratory and clinical features of SIC and hypofibrinolytic and hyperfibrinolytic DIC are presented in Table 1 [17].

The overall management of patients with SIC or DIC includes treatment of underlying causes and general ICU care. There is an increased risk of thrombosis as well as bleeding. With prolonged PT and aPTT, fresh frozen plasma (FFP) is administered if there is a high risk of bleeding or active bleeding, not for any specific values of PT or aPTT. Factor replacement may be indicated if FFP is unavailable or cannot be administered because of concerns of volume overload. Importantly, factor replacement does not correct all the factors depleted during the DIC’s global consumption process. Therapeutic plasma exchange (TPE) reduces circulating TF and potentially benefits patients with sepsis-induced DIC [32]. TPE has been shown to benefit adults with sepsis [33,34,35,36,37,38]. The benefit of TPE may be due to the removal of the harmful mediators of sepsis and the replacement of the plasma factors consumed [32]. There is no substantial evidence for indications of platelet transfusions and at what platelet levels they are to be transfused. There are guidelines from various societies for specific clinical situations regarding indications and thresholds for platelet transfusions [39,40].

### 3.2. Thrombocytopenia-Associated Multiple Organ Failure (TAMOF)

TAMOF is defined by new-onset thrombocytopenia, multiple organ failure, and elevated LDH levels [41]. TAMOF includes a range of conditions characterized by a combination of microangiopathies and coagulopathies, such as TTP, hemolytic uremic syndrome (HUS), and DIC. Most children with TAMOF show signs of increased VWF-mediated thrombosis, resembling the pathophysiology of TTP, although they do not have the classic form of TTP [42]. A majority of critically ill children with TAMOF have acquired ADAMTS13 deficiency [43]. Several other factors that occur during sepsis and inflammation can inhibit ADAMTS13 activity [42]. Deficiency of ADAMTS13 prevents the breakdown of large multimers of VWF released by the endothelium during inflammation and promotes platelet aggregation and microvascular thrombosis [42]. TAMOF is associated with low ADAMTS13 activity, organ failure, acute kidney injury (AKI), and thrombocytopenia [44,45,46]. Carcillo et al. used ADAMTS13 activity levels < 57% compared to normal as a threshold to identify TAMOF in classifying sepsis phenotypes [44,45]. An analysis of the Phenotyping Pediatric Sepsis-induced Multiple Organ Failure (PHENOMS) study subjects revealed an incidence of TAMOF > 20% [44,47].

The presence of TAMOF should trigger the clinician to evaluate the VWF/platelet pathway with ADAMTS13 and VWF, the fibrin pathway with a DIC panel, and the complement pathway for complement-mediated TMA. As in TTP, children with sepsis associated with low platelets and low ADAMTS13 without overt evidence of DIC or TTP may respond to TPE [43]. Several new therapeutic interventions are being attempted for TTP, which may benefit patients with TAMOF. Recombinant ADAMTS13 has been trialed for congenital TTP and anti-VWF nanobody for acquired TTP [42]. N-acetylcysteine is known to reduce the size of the VWF and has been used to treat refractory TTP [48]. Further studies are needed to determine their role in the management of TAMOF.

### 3.3. Thrombotic Microangiopathies (TMAs)

Thrombotic microangiopathies are characterized by thrombocytopenia, microangiopathic hemolytic anemia (MAHA), and organ damage in which ischemic organ injury may occur in the brain, kidneys, heart, pancreas, liver, lungs, eyes, and skin [18,49]. The features of MAHA include schistocytes on the peripheral blood smear, elevated LDH, low haptoglobin, elevated indirect bilirubin, and a decrease in baseline hemoglobin accompanied by thrombocytopenia [49].

TMA may present as HUS from Shiga toxin-producing Escherichia coli (STEC) infection, TTP, or complement-mediated TMA. An international consensus group has presented the etiology and pathophysiology-based classification of various forms of TMA [50]. In the past, HUS was classified as diarrhea-positive (D+ HUS) and diarrhea-negative (D- HUS or atypical HUS). There is an evolving understanding of what constitutes atypical HUS. STEC-HUS, pneumococcal-HUS, cobalamin C deficiency, TTP, and DIC should be ruled out before diagnosing atypical HUS (aHUS) [49,50].

Several conditions are associated with TMA, including transplantation (TA-TMA), malignancy (MA-TMA), pregnancy (HELLP syndrome), infections (EBV, CMV, HIV, etc.), and autoimmune diseases [49]. Additionally, some drugs are associated with TMA (Table 2) [51]. Table 2 includes the list of drugs that are implicated in developing thrombotic microangiopathy [51].

Malignancies, sepsis, transplantation, systemic autoimmune disorders, etc., are associated with secondary TMA. Secondary TMA may be associated with relative ADAMTS13 deficiency, inflammation, and complement activation through classical and alternative pathways [52]. Whether complement inhibition could be a possible therapeutic option for patients with secondary TMA is yet to be determined [52].

In cancer patients, TMA may be directly linked to the underlying malignancy, to chemotherapy, or may arise as an unrelated incidental condition. TPE is often ineffective in addressing many causes of TMA that are observed in cancer patients. When possible, treating the underlying malignancy is crucial for controlling both MA-TMA and DIC associated with cancer. Drug-induced TMA should be considered, and any suspected medications should be discontinued. Emerging evidence suggests that complement inhibition may play a role in managing some instances of drug-related TMA and TA-TMA in patients with malignancy [53]. Eculizumab may improve outcomes in TA-TMA [54,55].

Features of TMA are also observed with sepsis and DIC. Prolongation of coagulation tests, low fibrinogen levels, and high fibrin degradation products help to differentiate DIC from aTMA. A positive antiglobulin (Coombs) test may indicate immune thrombocytopenia, pneumococcal HUS, or Evans syndrome [49]. A high proportion of patients with atypical TMA (aTMA) or aHUS have underlying genetic abnormalities [49].

In acutely ill children, TMA should be suspected with the presence of MAHA, thrombocytopenia, and evidence of one or more organ dysfunction. The management of a child with suspected TMA includes obtaining a thorough history to evaluate the presence of any TMA-associated conditions. It is important to consider other conditions such as DIC, STEC-HUS, and pneumococcal-HUS and obtain laboratory tests for ADAMTS13 levels, cobalamin C deficiency, and complement activation. Along with supportive care and the treatment of underlying conditions, TPE is a reasonable treatment choice while awaiting the results of the ADAMTS13 levels and other test results. If TTP is ruled out, then eculizumab may be considered if there is a strong suspicion of complement-mediated TMA while waiting for genetic and other test results.

**Figure 3:**
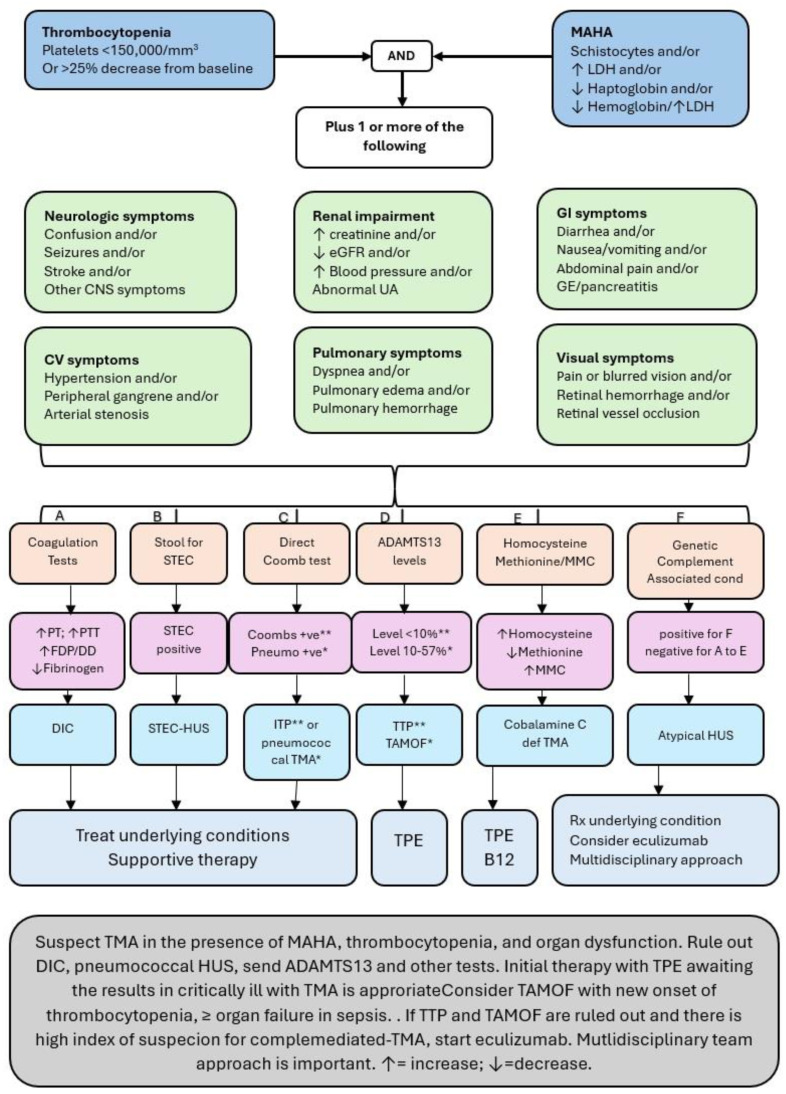
A summary of differential diagnoses, laboratory workups, and treatment choices in children with thrombocytopenia and microangiopathic hemolytic anemia. ** and * are matching conditions.

Several patients with TMA are admitted to ICUs during their initial presentations, and several conditions that are encountered are associated with the development of thrombotic microangiopathies. Early recognition and treatment are important to improve the outcomes in such cases [56]. Our understanding of TMA in acutely ill patients and the management strategies are rapidly evolving. A multidisciplinary team consisting of an intensive care physician, hematology/oncology, nephrology, and other specialties should be involved in their management. Figure 3 briefly summarizes the differential diagnoses, laboratory workups, and treatment choices [49,50].

#### DIC vs. TMA

Several laboratory abnormalities in patients with thrombocytopenia can be present in patients with DIC and TMA [57]. DIC is observed more frequently than TMA in critically ill patients [58]. However, DIC and TMA may coexist in a given patient. It is important to differentiate between these two conditions as there are specific therapies for certain TMA syndromes, and DIC is managed by treating underlying conditions. The evidence for MAHA, elevated LDH levels, and negative Coomb’s test may be present in patients with DIC and TMA. However, coagulation abnormalities, such as prolonged PT, PTT, low fibrinogen levels, and elevated fibrin degradation products, are observed with DIC. Hypertension is more often observed with TMA and hypotension with DIC [57]. Organ failure is commonly observed in both conditions. Respiratory failure and shock occur more frequently in DIC, whereas renal failure occurs similarly in DIC and TMA, and cerebral dysfunction occurs more commonly in TMA [58].

### 3.4. HLH/MAS/Cytokine Storm Syndromes (CSSs)

Pathologic immune activation and hyperinflammation can lead to a syndrome of HLH in genetically predisposed individuals (primary or familial HLH) or in patients with autoinflammatory/immune conditions, malignancies, sepsis, metabolic diseases, iatrogenic immunosuppression, and other inflammatory conditions (secondary HLH) [59]. HLH/MAS should be considered in the differential diagnoses of any acutely ill child presenting with thrombocytopenia [15]. HLH is diagnosed with the presence of at least five of the following criteria: fever (>38.5 °C for 7 days), hypertriglyceridemia, cytopenia (at least two of three cell lines: hemoglobin, WBCs, or platelets), hypofibrinogenemia, hemophagocytosis (in the spleen, liver, bone marrow, lymph nodes, or other tissues), low or absent natural killer (NK) cell activity, hyperferritinemia (>500 µg/L), or elevated soluble CD25 (soluble IL-2 receptor) levels (>2400 U/mL) [60]. The H-score has been developed and validated in patients with secondary HLH (sHLH) with sepsis and malignancies [61], and it has shown discriminant value in pediatric and adult patients [62,63].

MAS is used for features of HLH with massive hyperinflammation in patients with juvenile idiopathic arthritis [64,65]. The criteria for diagnosis and classification of MAS secondary to various autoimmune diseases vary based on the different laboratory abnormalities associated with each [15,66,67,68,69,70]. The criteria proposed for the diagnosis of MAS in autoimmune conditions include ferritin levels > 684 ng/mL plus any two of the four additional features, platelet count ≤ 181 × 10^9^/L, AST > 48 U/L, triglycerides > 156 mg/dL, and fibrinogen ≤ 360 mg/dL [69].

Sepsis-induced HLH/MAS has been recognized with evidence of hyperinflammation (hyperferritininemia, high CRP, etc.) and features of DIC and hepatobiliary disease (HBD). Carcillo et al. defined sepsis-induced MAS with ferritin > 500 ng/mL and hepatobiliary disease (ALT > 100 U/L AND bilirubin > 1 mg/dL OR INR > 1.5) and features of DIC (platelets < 100 × 10^9^/L AND INR > 1.5) [44,45]. The Hellenic Sepsis Study group used the term macrophage-activation-like syndrome (MALS) instead of secondary HLH/MAS because the tissue diagnosis for the presence of hemophagocytosis is rarely conducted in acutely ill patients [71]. MALS was diagnosed either with the presence of HLH through H-score or with the presence of DIC and HBD. In their study, 3–4% of those patients with sepsis had features of MALS, and the presence of MALS was associated with higher mortality [71].

HLH may manifest in patients with malignancy either at the time of initial presentation or during cancer therapy [72]. Malignancy-associated HLH (M-HLH) may be observed in up to 1% of patients with malignancies and is more common in adults than in children [72,73]. Hematologic malignancies are most commonly involved. Immune dysregulation from primary malignancy, immunosuppression from cancer chemotherapy, or concomitant infections, especially viral infections, may lead to HLH in patients with malignancies [72]. A finding of a high sIL-2R/ferritin ratio (>2) may indicate M-HLH in patients with malignancies [74]. The diagnosis of M-HLH may warrant the use of lymphocytic therapies (steroids and etoposide) before initiating specific cancer chemotherapy [15].

In a single-center study, among forty-three children with HLH, three (6%) had familial HLH, three (6%) had autoimmune disease, six (12%) had malignancies, while the remaining thirty-one (72%) were labeled as idiopathic [75]. The presence of features of DIC and hepatobiliary disease (HBD) was associated with a worse prognosis [75]. DIC was found to be a high-risk factor in another study [76].

The most common toxicity that occurs after chimeric antigen receptor (CAR) T cell therapy is cytokine release syndrome (CRS) [77]. CRS is a hyperinflammatory state that is elicited by CAR-T cell therapy [78]. In most patients with severe CRS, the laboratory markers align with the criteria for HLH/MAS, and, in most cases, the HLH/MAS symptoms subside as CRS resolves [79]. Tocilizumab and steroids are the most common medications used for the management of CRS [77,79].

The diagnosis and management of hyperinflammation conditions in acutely ill children are complex and require a multidisciplinary team approach. Five of the eight criteria of the International Histiocyte Society guidelines are required to diagnose HLH [60]. Many of these criteria are also present in MOF and MAS associated with sepsis [80]. Castillo and Carcillo suggest a cautious approach to using HLH chemotherapy in patients with sepsis who meet the HLH-2004 criteria [80]. An algorithmic approach with a multidisciplinary team of specialists is warranted with the availability of various biological and chemotherapy agents to manage hyperinflammation.

A consensus-based guideline has been published for early recognition, diagnosis, supportive care, and treatment of patients with HLH in ICUs [15]. The guidelines defined three grades of severity of patients with HLH admitted to an ICU [15]: mild with no organ dysfunction other than coagulation or hematologic manifestations; moderate with a pSOFA score of 2 or less in addition to coagulation or hematologic manifestations; severe is with a pSOFA score of >2 in addition to coagulation or hematologic manifestations. The guidelines also suggest treatment options based on the severity and etiology of HLH [15].

An RCT of adults with severe sepsis did not find anakinra to be beneficial [81]. However, in a post hoc analysis, severe sepsis patients with features of DIC and HBD demonstrated significant improvement in 28-day mortality with anakinra treatment [82]. In a case series, anakinra was used in eight patients with a diagnosis of secondary HLH admitted to a PICU; seven of them survived hospitalization [83].

**Figure 4:**
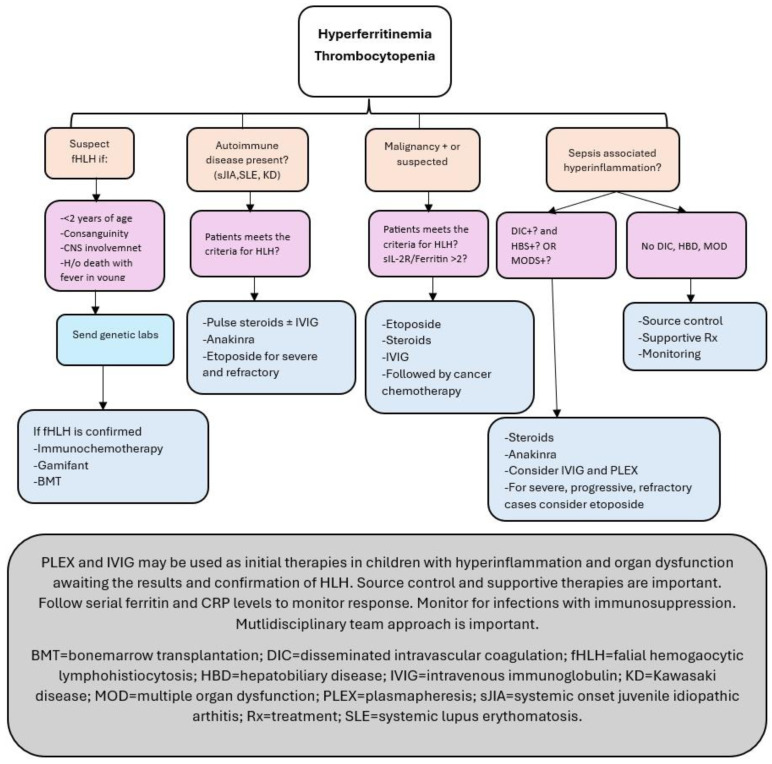
Diagnostic and therapeutic algorithms in children with thrombocytopenia and hyperferritinemia.

Our understanding of the frequency and management of HLH/MAS in the critically ill is in the early stages. HLH/MAS presents with varying severity and is associated with various underlying disease processes. A personalized diagnostic and therapeutic approach is advocated [84,85]. Immunochemotherapy and hematopoietic stem cell transplantation have improved survival in children considerably with primary HLH [86]. Typical immunosuppressive therapy for any patient meeting the criteria for HLH may be detrimental, and the outcome can be improved with an individualized approach [80,87,88,89]. Hyperferritinemia has a prognostic value in children with sepsis [90]. Using the clinical history and laboratory features of high ferritin levels, DIC, and HBD, the following diagnostic and therapeutic algorithm may be used based on the current understanding of hyperinflammatory conditions presenting as critical illness (Figure 4) [15,45,69,76,83,89]. Our approach to children with hyperferritinemia admitted to an ICU will change based on the results of several prospective trials that are ongoing [91].

### 3.5. Immune Thrombocytopenias

#### 3.5.1. Drug-Induced Immune Thrombocytopenia

Drugs can lead to thrombocytopenia by various mechanisms, including myelosuppression (as observed with chemotherapeutic agents), dose-dependent reduction in platelet production (such as with linezolid), or drug-induced immune thrombocytopenia (DITP) [92]. DITP occurs when drug-dependent antibodies attach to platelets, often causing severe thrombocytopenia that can result in life-threatening bleeding [92]. In a systematic evaluation of 153 drugs that were clinically implicated in thrombocytopenic reaction, 16 drugs met the criteria for a definite laboratory diagnosis of DITP [93]. These included quinine, quinidine, trimethoprim/sulfamethoxazole, vancomycin, penicillin, rifampin, carbamazepine, ceftriaxone, ibuprofen, mirtazapine, oxaliplatin, suramin, the glycoprotein Iib/IIIa inhibitors (abciximab, tirofiban, and eptifibatide), and heparin [93].

For a definite diagnosis of DITP to be established, the implicated drug has to be started before thrombocytopenia has developed, the platelet counts improve after stopping the offending medication, other causes of thrombocytopenia are excluded, and re-exposure to the medication leads to thrombocytopenia [92]. The level of evidence for DITP is classified based on how many criteria are met [92].

Several immune mechanisms, including drug-specific antibodies, autoantibodies, and immune complexes, are implicated in the genesis of DITP [92]. Typically, the patient is on the implicated drug for a week or longer before thrombocytopenia develops [92]. After stopping the offending medication, the symptoms of thrombocytopenia improve within a day or two, with the normalization of the platelet count in a week [92]. For patients in the ICU, drug-induced thrombocytopenia should be suspected if the platelet count decreases when the patient’s systemic condition improves.

The management of DITP includes stopping the offending agents. Corticosteroids are often used in DITP, but the evidence for their benefits is not strong. TPE and IVIG have been used in immune DITP with severe symptoms [92]. Once we establish that a drug is causing DITP, sensitivity for that drug probably persists indefinitely. Therefore, patients should be advised to avoid the medication thought to cause thrombocytopenia permanently [92].

#### 3.5.2. Heparin-Induced Thrombocytopenia (HIT)

Heparin-induced thrombocytopenia (HIT) is a life-threatening disorder that follows exposure to unfractionated or low-molecular-weight heparin. Patients classically present with a low platelet count (<150,000 per cubic mm) or a relative decrease of 50 percent or more from the baseline [94]. HIT is typically divided into type I and type II. Type I HIT, also called heparin-associated thrombocytopenia, is a non-immunologic reaction to heparin. A direct interaction between heparin and circulating platelets leads to platelet aggregation, clumping, and sequestration, which results in heparin-associated thrombocytopenia. In contrast, type II HIT is immune-mediated and is associated with an increased risk of thrombosis [95,96]. The antibodies against platelet factor 4 (PF4) and heparin complexes are responsible for causing type II HIT [94]. Thrombotic complications develop in approximately 20 to 50 percent of those patients with type II HIT [94]. Type I HIT is not associated with an increased risk of thrombosis [95]. HIT manifests approximately 5–10 days after starting heparin and earlier in patients with past exposure to heparin. HIT is a rare condition in children [96,97]. Meanwhile, type I HIT occurs early, within 48–72 h after heparin exposure [98].

The pre-test clinical score (4T-score) helps to assess the risk of HIT [99]. The laboratory tests include immune assays and functional assays for HIT antibodies. Because the results of laboratory tests for heparin antibodies are not immediately available, a clinical decision to discontinue heparin therapy should be undertaken based on the 4T-score pre-test probability. The management of HIT requires immediate cessation of administration of heparin and provision of alternate anticoagulation to prevent thrombosis once HIT is suspected [95]. Alternative anticoagulation therapy involves direct thrombin inhibitors such as argatroban, bivalirudin, or factor Xa inhibitors such as danaparoid and fondaparinux [95].

### 3.6. Dilutional Thrombocytopenia

Dilutional coagulopathy and dilutional thrombocytopenia can occur after resuscitation with large volumes of crystalloids and plasma and platelet-poor blood transfusions [100,101]. This can occur after trauma, massive bleeding, or major surgical procedures.

#### 3.6.1. Postoperative Thrombocytopenia

##### Cardiac Surgery

Thrombocytopenia can occur after cardiopulmonary bypass from hemodilution, platelet destruction, and platelet activation [102,103]. Typically, the nadir of the platelet count develops 48–72 h after CPB. In a cohort of adult patients after cardiopulmonary bypass (CPB), more than half developed thrombocytopenia to <125 × 10^9^/L, and 85% recovered their platelet counts at discharge [104]. In another study, thrombocytopenia (defined as <75 × 10^9^/L within 72 h) occurred in 26% of the adults after CPB, and, in a multivariable analysis, platelet nadir was associated with mortality [105]. Although it is uncommon, persistent thrombocytopenia after CPB may be due to HIT and is associated with worse outcomes [106]. HIT after CPB is rare in children but can occur [107].

In children and neonates, CPB is associated with thrombocytopenia, platelet dysfunction, and coagulopathy [108,109,110,111,112,113,114,115]. CPB is associated with thrombocytopenia and decreased platelet responsiveness [116]. The incidence of thrombocytopenia after cardiac surgery may be higher in children with trisomy 21 [117]. In a prospective study of 22 children under 4 years of age, CPB resulted in thrombocytopenia and prolonged PT and PTT [108]. The nadir of the platelet count occurred 48 h after cardiac surgery, and the nadir was associated with the duration of CPB [108]. Platelet and fibrinogen levels are decreased during CPB, and transfusion of platelets and cryoprecipitate normalizes these values after cardiac surgery [118].

##### Scoliosis Surgery

Scoliosis surgery is associated with significant blood loss and transfusion requirements [119]. Dilutional coagulopathy is a contributor to blood loss, as shown in a study where evidence of coagulopathy was present with increased fluid administration [120]. Platelet dysfunction, coagulation activation, and fibrinolysis may contribute to bleeding, and antifibrinolytics may help to decrease the transfusion requirements [121,122,123].

As per the Cochrane Database of Systemic Reviews, there is insufficient evidence for administering preprocedural prophylactic platelet transfusions in adults as there was a lack of evidence that transfusion resulted in a reduction in postoperative bleeding or all-cause mortality [124].

### 3.7. Distributional Thrombocytopenia

Distributional thrombocytopenia is attributed to the sequestration of platelets in patients with splenic enlargements such as cirrhosis or portal hypertension [125]. In patients with cirrhosis, thrombocytopenia may be due to reduced thrombopoietin in addition to distributional thrombocytopenia [125]. In patients with enlarged spleens, 80–90% of the transfused platelets may be trapped in the spleen [126]. Distributional thrombocytopenia is uncommon in acutely ill children but can occur in children with enlarged spleens from various causes.

Large hemangiomas and other vascular malformations may be associated with thrombocytopenia [127,128]. Thrombocytopenia from trapped platelets and consumptive coagulopathy can occur in patients with Kasabach–Merrit syndrome [127]. In most cases with distributional or consumptive thrombocytopenia, platelet transfusions do not lead to substantial increases in platelet counts and are reserved for acute life-threatening situations.

### 3.8. Extracorporeal Life Support and Thrombocytopenia

Thrombocytopenia is common in patients on ECMO support, irrespective of the type of ECMO [129,130]. The origin of thrombocytopenia in these patients is likely multifactorial. Underlying disease process, activation of platelets and coagulation with exposure to artificial surfaces, dilutional coagulopathy with the addition of a large volume circuit in neonates, HIT, and shear forces (platelet damage/destruction) via the ECMO circuit may contribute to thrombocytopenia and platelet dysfunction. In adults on ECMO support, the platelet count decreased by 7%, 35%, and 41% within 1, 24, and 48 h after initiation of ECMO support, respectively [131]. Shear-force-induced platelet clearance is thought to be the primary mechanism in this study for platelet reduction [131].

### 3.9. Trauma-Induced Coagulopathy

Trauma-induced coagulopathy (TIC) is common in children, with an incidence of 22% at the time of admission and 35% within 72 h [132]. Decreased platelet count is the second most common coagulation abnormality after prolonged PT in these children [132]. The presence of coagulopathy after trauma is associated with increased mortality [132]. Massive fluid resuscitation can worsen TIC, leading to a worse prognosis. Recent changes in the practice to mitigate the complications from TIC include minimizing fluid resuscitation with crystalloids, accepting permissive hypotension in certain situations, using fresh blood and antifibrinolytics, and balanced blood component transfusion [133,134]. Damage control resuscitation practices have improved the outcomes in adult trauma patients [135]. A transfusion ratio of >1.5:1 (RBC: platelets or FFP) is associated with a worse prognosis in adults [136]. Point-of-care testing and goal-directed coagulation therapy have been advocated [137,138,139,140].

## 4. Platelet Transfusion

Platelet transfusions are indicated in patients with thrombocytopenia or platelet dysfunction who are bleeding or at risk of bleeding. The platelet component types and thresholds for transfusion vary with the age of the patient, clinical condition, and the risk of bleeding [141].

Two main platelet unit types are generally available for transfusion [141]. Apheresis platelets are collected from single donors with automated cell separators. The typical platelet count in each single-donor platelet (SDP) unit is ≥3.0 × 10^11^ platelets per unit suspended in 100 to 500 mL of plasma or plasma plus platelet additive solution [141]. Typically, one SDP is used for an adult patient. The median volume of platelet transfusion in children is 9.4 mL/kg (IQR: 5.5–13.1) [142].

The other platelet type that is available for transfusion is a random-donor platelet (RDP) unit separated from the whole blood unit. Each RDP unit contains ≥5.5 × 10^10^ platelets per bag in 40 to 70 mL of plasma [141]. For adult patients, pooled platelets from 4 to 10 RDPs will be required for an adequate response [141].

Platelets are stored at room temperature (22 °C–24 °C) in gas-permeable bags [143]. They are stable for transfusion for 5 days after collection [143]. The shelf life may be extended to 7 days if the unit is tested negative for bacteria using a Food and Drug Administration-approved device. Storing platelets at room temperature can activate them, leading to metabolic and structural changes, platelet storage lesions, and increased microbial contamination [143]. Cold storage solutions are being investigated to mitigate the problems associated with room-temperature storage [143].

### 4.1. Blood Group Compatibility

Donor plasma should be ABO-compatible with the recipient’s blood, especially for neonates and children [141]. RhD-negative patients should receive RhD-negativeve platelets when available, especially in women of child-bearing age [141]. For critically ill neonates and pediatric patients in the RhD-negative blood group, if an RhD-negative unit is not available, RhD-positive platelets may be administered [40]. The apheresis unit contains minimal RBCs (<0.001 mL). Rh immune prophylaxis may be unnecessary if RhD-positive platelets are administered to RhD-negative patients [141], and prophylactic anti-RhD may be considered in a female child [144].

### 4.2. Platelet Threshold for Transfusion

In a prospective study, 3.3% of all the patients in 82 pediatric ICUs received at least one platelet transfusion [142]. Most of the platelet transfusions (67%) were for prophylaxis, and, in 34% of the prophylactic transfusions, the platelet count was ≥50 × 10^9^ per L [142]. In a single-center study, the mean threshold for platelet transfusion in critically ill children was 29 × 10^9^/L and varied based on the patient’s clinical condition [145].

The American Red Cross, the American Association of Blood Banks (AABB), and various other associations have published guidelines for platelet transfusions, especially for adult patients [39,141,144,146,147]. The Transfusion and Anemia eXpertise Initiative—Control/Avoidance of Bleeding (TAXI-CAB) through the Pediatric Critical Care Blood Research Network (BloodNet) of the Pediatric Acute Lung Injury and Sepsis Investigators (PALISI) Network published guidelines for platelet transfusions in critically ill neonates and children [40].

The suggested platelet transfusion thresholds in stable preterm and low-birth-weight neonates are in the range of 20–50 × 10^9^/L [147]. The rate of platelet transfusions was lower with the restrictive platelet transfusion strategy compared to the liberal strategy without increasing the severity or prevalence of intraventricular hemorrhage in preterm babies [148]. In suspected cases of neonatal alloimmune thrombocytopenia (NAIT), platelets that are negative for the HPA-1a/5b antigen should be transfused [144]. If maternal platelets are transfused in neonates with NAIT, they should be washed, irradiated, and volume-reduced [141]. The suggested thresholds for platelet transfusions at various ages and clinical indications are presented in Table 3.

### 4.3. Platelet-Transfusion-Associated Risks

In general, transfusion-related complications are more common with platelet transfusion compared to transfusion of any other blood product [141]. Febrile reactions are the most common risk associated with platelet transfusion, occurring in 1 in 14 transfusions [39,150]. Allergic reactions are reported in 1 in 50 transfusions [151]. The risk of bacterial infection from transfused platelets is 1 in 75,000 transfusions [152]. Bacterial infection is the most frequent infectious complication associated with platelet transfusions, occurring in 1 per 107,000 distributed units [141]. Transfusion-related acute lung injury (TRALI) is reported to occur in 1 in 138,000 transfusions [153]. However, the overall incidence of TRALI is higher at 1 in 10,000 transfusions for all plasma products [154]. In critically ill children, platelet transfusions are associated with increased mortality [142]. Children who require platelet transfusions have a higher acuity of illness, which may confound the association of mortality with platelet transfusion.

### 4.4. Platelet Refractoriness

The response to platelet transfusion is measured by count increment (CI) in the platelets within 10 to 60 min after the transfusion [141]. In adults, 1 RDP leads to CI of 5–10 × 10^9^/L of platelets and 60–100 × 10^9^/L per SDP [141]. In neonates and infants, 5–10 mL/kg of SDP should increase the platelet count by 50 to 100 × 10^9^/L [141]. Corrected Count Increase (CCI) is calculated as follows [141]:

CCI = CI × body surface area (m^2^)/number of platelets transfused (×10^11^).

Platelet refractoriness is defined as CCI less than 7.5 × 10^9^/L for two sequential transfusions [141]. Refractiveness of platelet transfusion may be due to immune or non-immune causes [141,149]. Alloimmunization to HLA antigen or uncommonly to human platelet antigen (HPA) should be investigated. The consumptive process may also lead to platelet refractoriness. In the absence of a consumptive process, HLA-matched platelet transfusions should be considered for platelet refractoriness [141]. A summary of the management of various clinical conditions with thrombocytopenia is presented in Table 4.

## 5. Future Directions

Our understanding of the various clinical syndromes with thrombocytopenia, such as various types of TMAs, TAMOF, and HLH, etc., is improving. However, early clinical identification of clinical phenotypes and their management still needs to be worked out. Prospectively collected registry data might be helpful in improving the identification and timely management of these conditions.

The evidence for the use of anti-thrombolytics in the management of DIC is mostly in adults. More research is needed regarding the identification of hyperfibrinolytic DI in children and the use of anti-fibrinolytics to understand the risks and benefits of such treatment. More research is needed to evaluate the value of thromboelastography in thrombocytopenic conditions in children.

Lastly, the recommendations for the threshold for platelet transfusion are mostly based on consensus. Reduced use of platelet transfusions and alternate strategies for the management of thrombocytopenia are other areas for future research.

## 6. Conclusions

Thrombocytopenia is relatively common in critically ill children. Diverse pathophysiologic mechanisms and etiologies can lead to thrombocytopenia in children. The presence of thrombocytopenia may signify a benign transient condition to a serious condition. The timing of occurrence of thrombocytopenia, the degree of thrombocytopenia, and associated clinical features in a given child will dictate the investigation of pathophysiologic causes and management. There is an increasing understanding of the mechanisms of thrombocytopenia and new therapeutic approaches for the management of specific conditions with thrombocytopenia. This review summarizes the various clinical conditions with thrombocytopenia and their management.

## Figures and Tables

**Table 1:** Clinical and laboratory features of sepsis-induced coagulopathy and overt DIC, hypofibrinolytic, and hyperfibrinolytic [17].

Plasma/Clinical Markers	Non-Overt DIC (SIC)	Overt DIC	
		Hypofibrinolytic	Hyperthrombolytic
Procoagulant marker			
Platelets	↓	↓↓	↓↓
Fibrinogen	↔/↑	↔/↓	↓↓↓
Tissue Factor	↑↑	↑↑↑	↑↑↑
TAT	↑↑	↑↑	↑↑↑
Anticoagulant markers			
Antithrombin III	↓↓	↓↓↓	↓↓↓
Protein C	↓↓	↓↓↓	↓↓↓
Thrombomodulin	↓↓	↓↓	↓↓↓
TFPI	↓↓	↓↓	↓↓
Antifibrinolytic markers			
PAI-1	↑↑	↑↑	↑↑
Fibrinolytic markers			
FDP/D-dimer	↔/↑	↑↑	↑↑↑
tPA	↔/↑	↔/↑	↑↑↑
Coagulation tests			
PT	↔	↑/↑↑	↑↑
aPTT	↔	↑/↑↑	↑↑
Clinical effects			
Thrombosis	↔/↑	↑↑	↔/↑
Bleeding	↔/↑	↔/↑	↑↑↑

↔ = no change, ↑, ↓ = mild changes, ↑↑, ↓↓ = moderate changes, and ↑↑↑, ↓↓↓ = severe changes. aPTT, activated partial thromboplastin time; DIC, disseminated intravascular coagulation; FDP, fibrin degradation product; PAI-1, plasminogen activator inhibitor-1; PT, prothrombin time; SIC, sepsis-induced coagulopathy; TAT, thrombin antithrombin complex; TFPI, tissue factor pathway inhibitor; tPA, tissue plasminogen activator.

**Table 2:** The list of drugs implicated in developing thrombotic microangiopathy [51].

Immune-Mediated TMA	Dose-Dependent TMA
Quinine	Cyclosporine
Oxaliplatin	Tacrolimus
Gemcitabine	Interferons: alpha, beta, and polycarboxylate
Muromonab-CD3	Sirolimus
Penicillin	Gemcitabine
Quetiapine	Bevacizumab
Sulfisoxazole	Mitomycin
Trielina	Pentostatin
Vancomycin	Sunitinib
	Cocaine
	Docetaxel
	Everolimus
	Vincristine

TMA = thrombotic microangiopathy.

**Table 3:** Suggested recommendations for the thresholds for platelet transfusions with source references.

Clinical Condition	Platelet Threshold (×10^9^/L)
Neonates	
Without bleeding	<25 [144] <20 [141]
Stable preterm without bleeding	<30 [141]
With bleeding or coagulopathy	<50 [144] <30 (Term) [141] <50 (preterm) [141]
With bleeding or major surgery (e.g., neurosurgery)	<100 [144]
Children	
Without complications (excluding immune thrombocytopenias)	<10 [40,144,149]
Aplastic anemia	<5 [141]
Mucositis	<20 [144]
Sepsis	<20 [144] <10 (no bleeding) [40] <50 (with bleeding) [40]
Moderate bleeding	<50 [144]
Major bleeding or significant postoperative bleeding	<75–100 [144]
ECMO	<80–100 [141]
Procedures	
Untunneled CVL	<20 [144]
Bone marrow biopsy	<10–20 [141] <20 [149]
Bronchoscopy or GI endoscopy	<20–50 [141]
Lumbar puncture	<40 [144] <20–50 [40]
Tunneled CVL	<50 [144]
Surgery	<50 [144] <20–50 [40]
Major surgery (e.g., neurosurgery)	<75–100 [144]
Spinal and epidural anesthesia	<80 [141]
Neuro-ophthalmic surgery	<100 [141]
ICP monitor	<100 [141]
Trauma	
Acute bleeding	<50 [141]
Multiple trauma/CNS bleeding	<100 [141]
Immune thrombocytopenia	
HLA alloimmunization	HLA-matched platelets [141]
ITP with life-threatening bleeding	High-dose platelets, IVIG, steroids [141]
TTP/HUS and HIT	Transfuse only for life-threatening bleeding or major invasive procedure [141]
NAIT	Maternal platelets, washed, irradiated [141] HPA-1a/5b-negative [141,144]

CNS: central nervous system; CVL: central venous line; ECMO: extracorporeal membrane oxygenation; HIT: heparin-induced thrombocytopenia; HLA: human leukocyte antigen; HPA: human platelet antigen; ICP: intracranial pressure; ITP: idiopathic thrombocytopenia; NAIT: neonatal alloimmune thrombocytopenia; TTP/HUS: thrombotic thrombocytopenic purpura/hemolytic uremic syndrome.

**Table 4:** Summary of management of various conditions with thrombocytopenia.

Clinical Condition	Platelet Transfusion	Other Potential Therapies
Spurious thrombocytopenia	Not indicated	Repeat measurement Visual inspection of the smear and manual platelet counting
Dilutional thrombocytopenia	Indicated in massive transfusion conditions; as clinically indicated	Prevention using massive transfusion protocols where clinically indicated
Decreased platelet production (bone marrow failure)	As clinically indicated	Diagnosis and management of underlying conditions leading to bone marrow failure
Sequestration of platelets (Splenic and portal system)	As clinically indicated Platelet resistance may occur	Splenectomy
Disseminated intravascular coagulation (DIC)	As clinically indicated	Treatment of underlying conditions In hyperfibrinolytic DIC: factor replacement and anti-fibrinolytic agents
Thrombocytopenia-Associated Multiple Organ Failure (TAMOF)	As clinically indicated	Treatment of underlying conditions TPE
TMA	Avoid platelet transfusions unless the risk of bleeding severe in all TMAs	
HUS-STEC		Supportive therapy Avoid antibiotics
TMA-pneumococcal		Supportive therapy Treat infection
TA-TMA Drug-induced TMA		Supportive therapy Remove offending drug Eculizumab
MA-TMA		Supportive therapy Eculizumab/TPE usually not effective
Cobalamin def TMA		B12; TPE
Atypical TMA		Supportive therapy Eculizumab—consider
TTP		TPE
ITP	Avoid platelet transfusions	Steroids; IVIG; DDAVP
fHLH	As clinically indicated	Immunochemotherapy Gamifant BMT
sHLH	As clinically indicated	Pulse steroids ± IVIG Anakinra Etoposide Treat underlying conditions
DITP	As clinically indicated	Remove the offending drug Observation For immune-mediated DITP: Steroids; IVIG; TPE
HIT	Typically not needed	Stop heparin Start direct thrombin inhibitors for anticoagulation

DITP: drug-induced thrombocytopenia; HIT: heparin-induced thrombocytopenia; fHLH: familial hemophagocytic lymphohistiocytosis; sHLH: secondary hemophagocytic lymphohistiocytosis; HUS: hemolytic uremic syndrome; ITP: immune thrombocytopenia; TMA: thrombotic microangiopathy; MA-TMA: malignancy-associated TMA; TA-TMA: transplant-associated TMA; TTP: thrombotic thrombocytopenic purpura.
